# Phages in the Human Body

**DOI:** 10.3389/fmicb.2017.00566

**Published:** 2017-04-04

**Authors:** Ferran Navarro, Maite Muniesa

**Affiliations:** ^1^Servei de Microbiologia, Hospital de la Santa Creu i Sant Pau, Institut d’Investigació Biomèdica Sant PauBarcelona, Spain; ^2^Department of Microbiology, University of BarcelonaBarcelona, Spain

**Keywords:** bacteriophages, human biomes, homeostasis, metagenomics, diagnosis, virome

## Abstract

Bacteriophages, viruses that infect bacteria, have re-emerged as powerful regulators of bacterial populations in natural ecosystems. Phages invade the human body, just as they do other natural environments, to such an extent that they are the most numerous group in the human virome. This was only revealed in recent metagenomic studies, despite the fact that the presence of phages in the human body was reported decades ago. The influence of the presence of phages in humans has yet to be evaluated; but as in marine environments, a clear role in the regulation of bacterial populations could be envisaged, that might have an impact on human health. Moreover, phages are excellent vehicles of genetic transfer, and they contribute to the evolution of bacterial cells in the human body by spreading and acquiring DNA horizontally. The abundance of phages in the human body does not pass unnoticed and the immune system reacts to them, although it is not clear to what extent. Finally, the presence of phages in human samples, which most of the time is not considered, can influence and bias microbiological and molecular results; and, in view of the evidences, some studies suggest that more attention needs to be paid to their interference.

## Introduction

Bacteriophages were discovered in the second decade of the 20th century (Twort, 1915; D’Herelle, 1917). It was initially suggested the idea they could be used to lyse pathogenic bacteria as a treatment of infectious diseases. However, the idea was rapidly abandoned in western countries due to the introduction of antibiotics. For decades, phages have been the most common model entities for the study of viruses and their replication cycles. Studies of certain model phages have contributed significantly to the advancement of molecular biology, for example in identifying the basis of genetic material, as the code of nucleotide triplets of individual amino acids (Crick et al., 1961), and the restriction enzymes (Dussoix and Arber, 1962). Moreover, the first sequenced genome was that of an *Escherichia coli* phage: ϕX174 (Sanger et al., 1977). For some years, the interest for phages was limited to ecological studies and proposals for their use as indicators of fecal pollution (IAWPRC Study Group on Health Related Water Microbiology, 1991; Jofre et al., 2016); in general, bacteriophages have deserved less interest in comparison to their bacterial hosts or to animal viruses.

Nevertheless, the remarkable estimated number of 10^31^ phages on the Earth (Suttle, 2005) is commonly used by researchers to highlight the importance of phages, which are believed to outnumber any other class of biological entity on the planet. Phages have recently re-emerged as powerful regulators of the bacterial populations in natural ecosystems (Fuhrman, 1999). Moreover, because of the appearance of resistances to different antimicrobial agents, their potential use as antimicrobials has been revisited (Reardon, 2014). Most significantly, recent metagenomes describe the abundance of viral sequences both outside and inside bacterial cells. This highlights their ubiquity as mobile genetic elements that contribute and affect bacterial evolution, causing the emergence of new bacterial pathogens, mobilizing genes outside the cells, and other functions. Interest in metagenomes includes the study of human microbiomes, where phages again appear as extremely abundant and diverse elements. Researchers are only now starting to suspect that phages actively contribute to the homeostasis of the bacterial flora (De Paepe et al., 2014). Because many studies focus on the role of the human symbiotic microbiota in our wellness, phages thus appear as contributing actors that are directly related with human health (Manrique et al., 2016), and therefore the interest in them is rising.

## Phages as a Part of Human and Animal Microbiota

**FIGURE 1:**
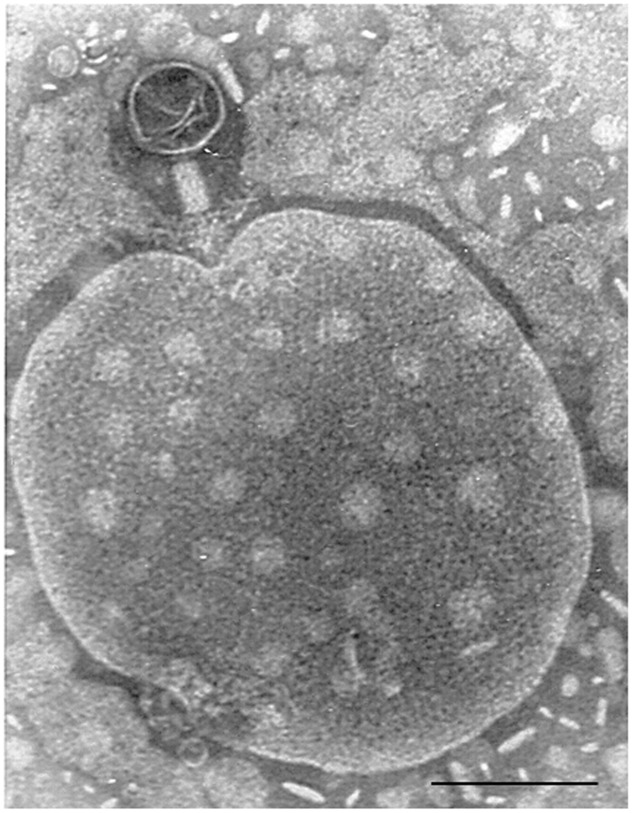
**Bacteriophage of *Myoviridae* morphology isolated from a fecal sample, attached to an unidentified particle.** Bar 100 nm.

Many metagenomic analyses of human microbiomes show the abundance of phages, which is generally greater than that of eukaryotic viruses. This has been shown in metagenomic analysis of lung, vaginal, skin, oral or intestinal microbiota (Breitbart et al., 2003; Colomer-Lluch et al., 2011a; Minot et al., 2011; Oh et al., 2014; Virgin, 2014). More recently, infectious phages have been found in different clinical samples such as ascitic fluid and urine (Brown-Jaque et al., 2016). It was suggested that they could reach the peritoneal cavity after translocation from the intestine (Górski et al., 2006), where they are present (**Figure 1**) and abundant. They are also present in voided urine (Brown-Jaque et al., 2016), probably coming from the periurethral area. In animals, phages infecting *Bacteroides* were found in serum (Keller and Traub, 1974), confirming their presence in the blood stream. Translocation of phages from blood to mouse fetal tissues has also been demonstrated in pregnant mice (Srivastava et al., 2004a).

In the light of these results, and as a second level of study, some researchers have analyzed solely the virome fraction of these microbiomes. To do this, they have devised methods that allow discrimination of the viral fraction, while discarding bacterial and free DNA. Those studies have yielded some surprising results; many viral particles in fact carry sequences identified as bacterial DNA. Shared genetic content is observed when analyzing the phage and bacterial DNA fractions of the same sample (Breitbart et al., 2003; Minot et al., 2011; Colombo et al., 2016; Howe et al., 2016), including sequences belonging to CRISPR-Cas systems (Dutilh et al., 2014).

CRISPR-Cas systems constitute a immune system that protect bacteria against bacteriophages and foreign DNA (Mojica and Rodriguez-Valera, 2016), that has later been applied for genome engineering in bacteria and eukaryotes. The different activity of the CRISPR-Cas systems influences the allowance of bacterial cells to foreign DNA or their immunity to phage infection, and this can shape the evolution of human microbiomes. Besides the use of CRISPR-Cas systems in genome engineering, the analysis of CRISPR sequences from raw metagenomic data has revealed unidentified phages, as crAssphage phage, that is claimed to be present in the majority of human fecal microbiomes, although it has never been isolated (Dutilh et al., 2014).

## Phages as Mobile Genetic Elements

Transduction, the process by which the DNA is mobilized between cell by a virus or viral vector was reported the last century (Zinder and Lederberg, 1952), although the rates of this mobilization has never been well defined. For this reason, the detection of an important proportion of bacterial DNA in phage particles observed in metagenomic analysis was indeed a surprise, and it initially prompted the belief that the methods for segregating phage and bacterial particles were not accurate enough, and either bacterial or free DNA contaminated the phage samples. However, the protocols have been optimized allowing specific extraction of packaged DNA. Another suspicion is that the bioinformatic analysis failed to identify phage DNA sequences correctly and they were mistaken for bacterial DNA. Nevertheless, subsequent repetitions and more accurate approaches have shown that despite some of these problems occurring, a relevant fraction of the virome is actually mobilizing bacterial DNA. This has led to the suspicion that bacterial cells use the numerous capsid genes that they possess, probably inherited from ancient prophage remnants, to build protein capsids that pack and spread their DNA content (Asadulghani et al., 2009; Lang et al., 2012; Penades et al., 2015).

The fact that phage capsids can mobilize bacterial DNA has multiple consequences, such as, for example, the fact that they can mobilize and transduce virulence genes (O’Brien et al., 1984; Griffiths et al., 2000; Allué-Guardia et al., 2011; Penades et al., 2015), antibiotic resistances (Muniesa et al., 2004; Colomer-Lluch et al., 2011b; Ross and Topp, 2015; Haaber et al., 2016) or genes related to fitness (Lindell et al., 2004; Müller et al., 2013) to new bacterial hosts. This causes horizontal genetic exchange and leads to the evolution of bacterial populations.

## Phages as Regulators of Populations

Bacterial populations can change and evolve through acquisition of new genes transferred by phages, but also by predation and lysis caused by phages. Experimental evidence from chemostats and observations of phages/hosts in open systems has shown that for some bacterial species, populations of phages and hosts oscillate over time, following a “Red Queen/kill-the-winner” dynamics,” which describes prey–predator variations (Rodriguez-Brito et al., 2010; Jover et al., 2013; Lim et al., 2015). However, phage–host dynamics can change in accordance with the homogeneity and structure of the environment, and also depending on the conditions that facilitate phage–cell encounters (De Paepe et al., 2014).

Changes or a total replacement of the microbiome by a fecal transplant in diseases without a well-defined etiological agent, such as inflammatory bowel diseases (Crohn’s disease or ulcerative colitis), can result in different disease outcomes (Loh and Blaut, 2012; Moayyedi et al., 2015). Comparison of the viromes of individuals suffering from Crohn’s disease and healthy relatives revealed differences in composition and variability (Pérez-Brocal et al., 2013; Wagner et al., 2013). Whether changes in the phagome of human biomes is a cause or a consequence of dysbiosis in such diseases has not yet been established. Considering that the phagome could influence bacterial populations, two options are plausible: changes in bacteria could cause variations in the distribution of phage groups; or changes in the phagome could be responsible of dysbacteriosis (Norman et al., 2015; Pérez-Brocal et al., 2015).

Similarly, phages have been detected in the metagenomes of sputum of patients suffering cystic fibrosis (Willner et al., 2009); and both the phage diversity and relative abundances were reported to be different from those of non-cystic fibrosis patients. It is hard, however, to conclude from these results what the cause of these differences is. Some variations in bacteria are caused by phages and those variations could be harmful to the patients. For example, mucoid isolates of *Pseudomonas fluorescens* are more virulent than their non-mucoid isogenic variants. This mucoid overproduction is a virulence factor contributing to more persistent infections in cystic fibrosis patients (Scanlan and Buckling, 2012). This phenotypic characteristic is favorably selected in the presence of phages, because it confers protection against phage infection. Accordingly, the mucoid isolates became resistant to the phages with the corresponding detrimental consequence for the patients (Scanlan and Buckling, 2012).

A different example of the regulation of human bacterial populations by phages is observed when we look at the competition between *Streptococcus pneumoniae* and *Staphylococcus aureus*. The former produces hydrogen peroxide; an agent that induces the bacterial SOS response and can induce temperate prophages. Meanwhile, the vast majority of *S. aureus* strains carry prophages that could be induced in the presence of the concentrations of H_2_O_2_ produced by *S. pneumoniae*. *S. pneumoniae* prophages, in turn, are not induced at these concentrations. The result is that *S. pneumoniae* prevails by killing *S. aureus* lysogenic strains via induction of prophages that cause the subsequent lysis of the cell (Selva et al., 2009).

Yet another example of how bacteriophages can impact the dynamics of bacterial populations has been observed in *Enterococcus faecalis* V583. This strain produces a composite phage ΦV1/7, derived from two distinct chromosomally encoded prophage elements. Prophage ΦV1 produces the capsids, while prophage ΦV7 is in charge of infection of susceptible hosts and V583 can produce infectious ΦV1/7. The induction of ΦV1/7 is highly enhanced by the availability of free amino acids in the medium. The strain producing ΦV1/7 has an advantage over other *E. faecalis* strains in the intestine, because these are lysed by ΦV1/7, while V583 is resistant to superinfection, enhancing the success of *E. faecalis* V583 during competitive growth (Duerkop et al., 2012).

## Interactions With the Immune System

It is not clear whether phages can easily be detected by the immune system, or whether they interact with it. Because the size of phage particles is usually bigger than eukaryotic viruses, activation of the immune system might occur as for other viruses. The desire to use phages to treat bacterial infections has led to explorations of the responses that phages might cause within the human immune system.

Very soon after the discovery of phages, it was observed that antibodies against bacteriophages in humans or animals were produced (Jerne, 1952, 1956); and it is easy to generate phage antiserums by immunization of humans or animals with phage lysates (Puig et al., 2001; Gorski et al., 2012; Bacon et al., 2017). The sera of non-immunized individuals (humans or animals) present antibodies against phages, although at low levels; the so-called “natural antibodies.” For instance, antibodies against T4 phages are naturally present in human serum (Dabrowska et al., 2014) presumably as a consequence of the confirmed constant presence of phages in human biomes (Górski et al., 2006; Brown-Jaque et al., 2016). However, the origin of natural antibodies, generally of IgM class, with broad cross reactivity and low affinity, is not clear in the majority of cases.

The innate immune system, particularly by the components of the reticuloendothelial system (RES), could be a mechanism for removing phages that are circulating in the human body (Gorski et al., 2012). Certainly, this system was credited with the rapid removal of administered wild-type phage λ from the circulatory system in humans (Geier et al., 1973). Moreover, different phage λ mutants could induce different host responses. When using certain phage λ mutants that were capable of circumventing the RES immune response, these mutants prevailed for longer periods in the blood stream than the wild-type phage (Merril et al., 1996).

Data on anti-phage cellular responses are very scarce in comparison with data on phage–humoral responses. The only study we are aware of, evaluated the cellular response to MS2 phage that was intradermally administrated in guinea pigs. The presence of the phage produced erythema and induration, that are signs of cell-mediated immunity (Langbeheim et al., 1978). In contrast, another study showed that the permanence of phages in blood is the same when comparing immunocompetent mice or those deficient in T-cells, indicating no specific role of T-cell response in phage inactivation (Srivastava et al., 2004b).

When administered together with the host bacteria, some studies showed that phages seem to stimulate bacterial phagocytosis, and this is attributed to certain “opsonization” of the bacterial cells by phages. In addition, phages can remain active and infective when adsorbed onto the bacteria on intake by granulocytes. Therefore, some authors have suggested that during phagocytosis, phages continue lysing the phagocytosed bacteria, helping the activity of phagocytic cells. This process is limited in time and phages are no longer active after the completion of phagocytosis (Gorski et al., 2012). Despite these descriptions, there is no definitive evidence that phages activate phagocytosis by themselves, and some years ago, a contrary outcome was reported (Kantoch et al., 1958). In those studies, when used at very high doses (10^10^/ml), phages inhibited phagocytosis of their host bacteria, and this inactivation was observed using either infectious or heat-inactivated phages (Kantoch et al., 1958). Inhibition was greater when using antibody-treated phages, and therefore the authors suggested that the immunocomplexes phage–antibody would be inactivating factors particularly active (Kantoch et al., 1958). Moreover, purified phages have anti-inflammatory effects via suppression of ROS (reactive oxygen species) production and inhibition of NF-κβ activity, affecting the production of cytokines [for a review, see (Gorski et al., 2012)]. Despite this evidence, it should be borne in mind that many experiments have been conducted with phage lysates, which on many occasions could contain remnants of bacteria lysed by the phages (e.g., lipopolysaccharide) or perhaps fragments of the host bacterial cell wall adhered to the phage tails. This makes it extremely difficult to determine the components truly responsible for the modulation of the immune response.

## Interference With Clinical Diagnoses

Assuming the relative occurrence and distribution of phages throughout the human body described above, coincident with the location of their bacterial hosts, and highly plausible translocation of phage particles to other areas of the body, some reports indicate that the neglected presence of phages in human samples could have an important influence by interfering in clinical practice (Brown-Jaque et al., 2016).

It has been shown that the presence of phages could interfere with many protocols intended to isolate bacteria by enrichment broth, since the phages in the sample destroy the bacterial cells during the enrichment procedure (Muniesa et al., 2005; Quiros et al., 2015). Phages might also interfere in clinical settings during bacterial isolation. As indicated above, there is evidence of a lack of, or reduced, bacterial isolation in clinical samples (ascitic fluid and urine) carrying a high titer of phages, because the lytic activity of the phages disturbs the isolation of the target bacteria (Brown-Jaque et al., 2016).

In addition, some results obtained using molecular methods when targeting some virulence genes present in pathogenic bacteria could be confusing. This is because some genes are located in temperate phages and DNA extraction methods do not distinguish between bacterial and phage DNA. This is the case for phages encoding the Shiga toxin gene, which can be detected in the absence of Shiga toxin-producing bacteria (Martínez-Castillo and Muniesa, 2014). Detection of certain bacterial groups by 16SrDNA qPCR or by genomic sequencing in mixed samples might also be confusing if the sample contains phages and the DNA in the phages is actually what is amplified in the absence of intact bacterial cells. This might be an explanation of the mismatch between the high number of gene copies of 16SrDNA obtained by qPCR amplification and the lack of bacterial isolation observed sometimes (Esparcia et al., 2011). Among others, one hypothesis could be that the positive results were due to amplification of bacterial DNA within phage particles or of bacterial DNA released in the sample after phage-mediated lysis.

## Use of Phages Against Human Bacterial Pathogens

The problems of fighting antibiotic resistance in bacteria are continually increasing and severely undermine our capacity to control bacterial infectious diseases. After the increased incidence of bacterial resistance to antibiotics over recent decades, phages have surfaced again as alternative or complementary therapies to control bacterial infections (Fischetti et al., 2006; Doyle and Erickson, 2012; Hertwig et al., 2013; Reardon, 2014; Schmelcher and Loessner, 2014; Górski et al., 2016).

## Concluding Remarks

**FIGURE 2:**
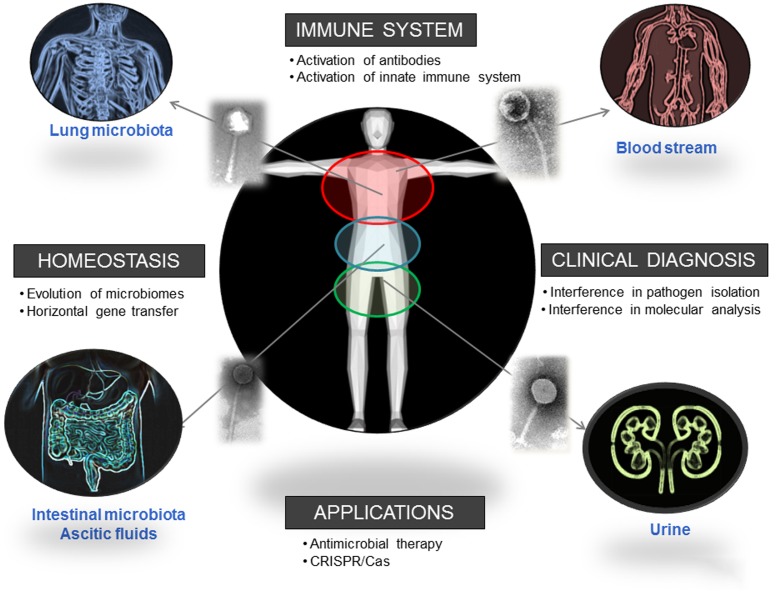
**Biomes where the presence of phages has been reported and direct and indirect ways in which phages influence human health.** Pictures has been adapted from Pixabay.

Phages, the most abundant entities on the planet, are also present in human biomes. This presence is known and recognized, but sometimes neglected; and it has a strong influence on the distribution and dynamics of different bacterial populations. Considering the influence of these populations in human health, as their reported ability to improve digestive health, it is clear that phages can be directly related with human well-being (**Figure 2**). The influence of phages in different mechanisms of our immune system suggests a long-term relationship that we are just starting to elucidate. Moreover, considering our interest in isolating and identifying bacterial pathogens, the presence of phages could certainly interfere with that analysis if it is not considered. A One Health multidisciplinary approach, not restricted to academic or clinical settings and not limited either to microbiological studies, is advisable to evaluate the real extent of and the role played by the phagome in human bodies.

## Author Contributions

All authors listed, have made substantial, direct and intellectual contribution to the work, and approved it for publication.

## Conflict of Interest Statement

The authors declare that the research was conducted in the absence of any commercial or financial relationships that could be construed as a potential conflict of interest.

## References

[B1] Allué-Guardia A. García-Aljaro C. Muniesa M. (2011). Bacteriophage-encoding cytolethal distending toxin type V gene induced from nonclinical *Escherichia coli* isolates. *Infect. Immun.* 79 3262–3272. 10.1128/IAI.05071-11 21646456 PMC3147592

[B2] Asadulghani M. Ogura Y. Ooka T. Itoh T. Sawaguchi A. Iguchi A. (2009). The defective prophage pool of *Escherichia coli* O157: prophage-prophage interactions potentiate horizontal transfer of virulence determinants. *PLoS Pathog.* 5:e1000408 10.1371/journal.ppat.1000408 PMC2669165 19412337

[B3] Bacon E. J. Richmond S. J. Wood D. J. Stirling P. Bevan B. J. Chalmers W. S. (2017). Serological detection of phage infection in *Chlamydia psittaci* recovered from ducks. *Vet. Rec.* 119 618–620. 3544465

[B4] Breitbart M. Hewson I. Felts B. Mahaffy J. M. Nulton J. Salamon P. (2003). Metagenomic analyses of an uncultured viral community from human feces. *J. Bacteriol.* 185 6220–6223. 10.1128/JB.185.20.6220-6223.2003 14526037 PMC225035

[B5] Brown-Jaque M. Muniesa M. Navarro F. (2016). Bacteriophages in clinical samples can interfere with microbiological diagnostic tools. *Sci. Rep.* 6:33000 10.1038/srep33000 PMC5016790 27609086

[B6] Colombo S. Arioli S. Guglielmetti S. Lunelli F. Mora D. (2016). Virome-associated antibiotic-resistance genes in an experimental aquaculture facility. *FEMS Microbiol. Ecol.* 92:fiw003 10.1093/femsec/fiw003 26738553

[B7] Colomer-Lluch M. Imamovic L. Jofre J. Muniesa M. (2011a). Bacteriophages carrying antibiotic resistance genes in fecal waste from cattle, pigs, and poultry. *Antimicrob. Agents Chemother.* 55 4908–4911. 10.1128/AAC.00535-11 21807968 PMC3187014

[B8] Colomer-Lluch M. Jofre J. Muniesa M. (2011b). Antibiotic resistance genes in the bacteriophage DNA fraction of environmental samples. *PLoS ONE* 6:e17549 10.1371/journal.pone.0017549 PMC3048399 21390233

[B9] Dutilh B. E. Cassman N. McNair K. Sanchez S. E. Silva G. G. Z. Boling L. (2014). A highly abundant bacteriophage discovered in the unknown sequences of human faecal metagenomes. *Nat. Commun.* 5 4498 10.1038/ncomms5498 PMC4111155 25058116

[B10] Crick F. H. Barnett L. Brenner S. Watts-Tobin R. J. (1961). General nature of the genetic code for proteins. *Nature* 192 1227–1232. 10.1038/1921227a0 13882203

[B11] Dabrowska K. Miernikiewicz P. Piotrowicz A. Hodyra K. Owczarek B. Lecion D. (2014). Immunogenicity studies of proteins forming the T4 phage head surface. *J. Virol.* 88 12551–12557. 10.1128/JVI.02043-14 25142581 PMC4248953

[B12] De Paepe M. Leclerc M. Tinsley C. R. Petit M.-A. (2014). Bacteriophages: an underestimated role in human and animal health? *Front. Cell. Infect. Microbiol.* 4:39 10.3389/fcimb.2014.00039 PMC3975094 24734220

[B13] D’Herelle F. (1917). sur un microbe invisible antagonist des bacilles disenterique. *C. R. Acad. Sci. Ser. D* 165 373–375.

[B14] Doyle M. P. Erickson M. C. (2012). Opportunities for mitigating pathogen contamination during on-farm food production. *Int. J. Food Microbiol.* 152 54–74. 10.1016/j.ijfoodmicro.2011.02.037 21474196

[B15] Duerkop B. A. Clements C. V. Rollins D. Rodrigues J. L. M. Hooper L. V. (2012). A composite bacteriophage alters colonization by an intestinal commensal bacterium. *Proc. Natl. Acad. Sci. U.S.A.* 109 17621–17626. 10.1073/pnas.1206136109 23045666 PMC3491505

[B16] Dussoix D. Arber W. (1962). Host specificity of DNA produced by *Escherichia coli*. II. Control over acceptance of DNA from infecting phage lambda. *J. Mol. Biol.* 5 37–49. 10.1016/S0022-2836(62)80059-X 13888713

[B17] Esparcia O. Montemayor M. Ginovart G. Pomar V. Soriano G. Pericas R. (2011). Diagnostic accuracy of a 16S ribosomal DNA gene-based molecular technique (RT-PCR, microarray, and sequencing) for bacterial meningitis, early-onset neonatal sepsis, and spontaneous bacterial peritonitis. *Diagn. Microbiol. Infect. Dis.* 69 153–160. 10.1016/j.diagmicrobio.2010.10.022 21251558

[B18] Fischetti V. A. Nelson D. Schuch R. (2006). Reinventing phage therapy: are the parts greater than the sum? *Nat. Biotechnol.* 24 1508–1511. 10.1038/nbt1206-1508 17160051

[B19] Fuhrman J. A. (1999). Marine viruses and their biogeochemical and ecological effects. *Nature* 399 541–548. 10.1038/21119 10376593

[B20] Geier M. R. Trigg M. E. Merril C. R. (1973). Fate of bacteriophage lambda in non-immune germ-free mice. *Nature* 246 221–223. 10.1038/246221a0 4586796

[B21] Gorski A. Miedzybrodzk R. Borysowski J. Dabrowska K. Wierzbicki P. Ohams M. (2012). Chapter 2–“Phage as a modulator of immune responses: practical implications for phage therapy,” in *Advances in Virus Research. Bacteriophages Part B*, eds Lobocka M. Szybalski W. (Sant Diego, CA: Academic Press), 41–72. 10.1016/B978-0-12-394438-2.00002-5 22748808

[B22] Górski A. Miêdzybrodzki R. Weber-Dąabrowska B. Fortuna W. Letkiewicz S. Rogóż P. (2016). Phage therapy: combating infections with potential for evolving from merely a treatment for complications to targeting diseases. *Front. Microbiol.* 7:1515 10.3389/fmicb.2016.01515 PMC5035766 27725811

[B23] Górski A. Wazna E. Dabrowska B.-W. Dabrowska K. Switała-Jeleñ K. Miedzybrodzki R. (2006). Bacteriophage translocation. *FEMS Immunol. Med. Microbiol.* 46 313–319. 10.1111/j.1574-695X.2006.00044.x 16553803

[B24] Griffiths A. J. Miller J. H. Suzuki D. T. Lewontin R. C. Gelbart W. M. (2000). “Gene transfer in bacteria and their viruses,” in *An Introduction to Genetic Analysis*, 7th Edn, ed. Freeman W. (New York, NY: W. H. Freeman).

[B25] Haaber J. Leisner J. J. Cohn M. T. Catalan-Moreno A. Nielsen J. B. Westh H. (2016). Bacterial viruses enable their host to acquire antibiotic resistance genes from neighbouring cells. *Nat. Commun.* 7:13333 10.1038/ncomms13333 PMC5103068 27819286

[B26] Hertwig S. Hammerl J. A. Appel B. Alter T. (2013). Post-harvest application of lytic bacteriophages for biocontrol of foodborne pathogens and spoilage bacteria. *Berl. Münch. Tierärztl. Wochenschr.* 126 357–369. 24199377

[B27] Howe A. Ringus D. L. Williams R. J. Choo Z.-N. Greenwald S. M. Owens S. M. (2016). Divergent responses of viral and bacterial communities in the gut microbiome to dietary disturbances in mice. *ISME J.* 10 1217–1227. 10.1038/ismej.2015.183 26473721 PMC5029215

[B28] IAWPRC Study Group on Health Related Water Microbiology (1991). Bacteriophages as model viruses in water quality control. *Water Res* 25 529–545. 10.1016/0043-1354(91)90126-B

[B29] Jerne N. K. (1952). Bacteriophage inactivation by antiphage serum diluted in distilled water. *Nature* 169 117–118. 10.1038/169117b0 14910703

[B30] Jerne N. K. (1956). The presence in normal serum of specific antibody against bacteriophage T4 and its increase during the earliest stages of immunization. *J. Immunol.* 76 209–216. 13306956

[B31] Jofre J. Lucena F. Blanch A. Muniesa M. (2016). Coliphages as model organisms in the characterization and management of water resources. *Water* 8 199 10.3390/w8050199

[B32] Jover L. F. Cortez M. H. Weitz J. S. (2013). Mechanisms of multi-strain coexistence in host-phage systems with nested infection networks. *J. Theor. Biol.* 332 65–77. 10.1016/j.jtbi.2013.04.011 23608631

[B33] Kantoch M. Skurski A. Wieczorek Z. (1958). In vitro blockade of bacterial phagocytosis of leukocytes by means of bacterial viruses. *Schweiz. Z. Pathol. Bakteriol.* 21 1106–1119. 10.1159/000160571 13624698

[B34] Keller R. Traub N. (1974). The characterization of *Bacteroides fragilis* bacteriophage recovered from animal sera: observations on the nature of *Bacteroides* phage carrier cultures. *J. Gen. Virol.* 24 179–189. 10.1099/0022-1317-24-1-179 4843748

[B35] Lang A. S. Zhaxybayeva O. Beatty J. T. (2012). Gene transfer agents: phage-like elements of genetic exchange. *Nat. Rev. Microbiol.* 10 472–482. 10.1038/nrmicro2802 22683880 PMC3626599

[B36] Langbeheim H. Teitelbaum D. Arnon R. (1978). Cellular immune response toward MS-2 phage and a synthetic fragment of its coat protein. *Cell. Immunol.* 38 193–197. 10.1016/0008-8749(78)90046-1 78768

[B37] Lim E. S. Zhou Y. Zhao G. Bauer I. K. Droit L. Ndao I. M. (2015). Early life dynamics of the human gut virome and bacterial microbiome in infants. *Nat. Med.* 21 1228–1234. 10.1038/nm.3950 26366711 PMC4710368

[B38] Lindell D. Sullivan M. B. Johnson Z. I. Tolonen A. C. Rohwer F. Chisholm S. W. (2004). Transfer of photosynthesis genes to and from *Prochlorococcus* viruses. *Proc. Natl. Acad. Sci. U.S.A.* 101 11013–11018. 10.1073/pnas.0401526101 15256601 PMC503735

[B39] Loh G. Blaut M. (2012). Role of commensal gut bacteria in inflammatory bowel diseases. *Gut Microbes* 3 544–555. 10.4161/gmic.22156 23060017 PMC3495792

[B40] Manrique P. Bolduc B. Walk S. T. van der Oost J. de Vos W. M. Young M. J. (2016). Healthy human gut phageome. *Proc. Natl. Acad. Sci. U.S.A.* 113 10400–10405. 10.1073/pnas.1601060113 27573828 PMC5027468

[B41] Martínez-Castillo A. Muniesa M. (2014). Implications of free Shiga toxin-converting bacteriophages occurring outside bacteria for the evolution and the detection of Shiga toxin-producing *Escherichia coli*. *Front. Cell. Infect. Microbiol.* 4:46 10.3389/fcimb.2014.00046 PMC3997033 24795866

[B42] Merril C. R. Biswas B. Carlton R. Jensen N. C. Creed G. J. Zullo S. (1996). Long-circulating bacteriophage as antibacterial agents. *Proc. Natl. Acad. Sci. U.S.A.* 93 3188–3192. 10.1073/pnas.93.8.3188 8622911 PMC39580

[B43] Minot S. Sinha R. Chen J. Li H. Keilbaugh S. A. Wu G. D. (2011). The human gut virome: inter-individual variation and dynamic response to diet. *Genome Res.* 21 1616–1625. 10.1101/gr.122705.111 21880779 PMC3202279

[B44] Moayyedi P. Surette M. G. Kim P. T. Libertucci J. Wolfe M. Onischi C. (2015). Fecal Microbiota transplantation induces remission in patients with active ulcerative colitis in a randomized controlled trial. *Gastroenterology* 149 102–109.e6. 10.1053/j.gastro.2015.04.001 25857665

[B45] Mojica F. J. M. Rodriguez-Valera F. (2016). The discovery of CRISPR in archaea and bacteria. *FEBS J.* 283 3162–3169. 10.1111/febs.13766 27234458

[B46] Müller M. G. Ing J. Y. Cheng M. K.-W. Flitter B. A. Moe G. R. (2013). Identification of a phage-encoded Ig-binding protein from invasive *Neisseria meningitidis*. *J. Immunol.* 191 3287–3296. 10.4049/jimmunol.1301153 23926326 PMC3780609

[B47] Muniesa M. Blanch A. R. Lucena F. Jofre J. (2005). Bacteriophages may bias outcome of bacterial enrichment cultures. *Appl. Environ. Microbiol.* 71 4269–4275. 10.1128/AEM.71.8.4269-4275.2005 16085813 PMC1183318

[B48] Muniesa M. García A. Miró E. Mirelis B. Prats G. Jofre J. (2004). Bacteriophages and diffusion of beta-lactamase genes. *Emerg. Infect. Dis.* 10 1134–1137. 10.3201/eid1006.030472 15207070 PMC3323147

[B49] Norman J. M. Handley S. A. Baldridge M. T. Droit L. Liu C. Y. Keller B. C. (2015). Disease-specific alterations in the enteric virome in inflammatory bowel disease. *Cell* 160 447–460. 10.1016/j.cell.2015.01.002 25619688 PMC4312520

[B50] O’Brien A. D. Newland J. W. Miller S. F. Holmes R. K. Smith H. W. Formal S. B. (1984). Shiga-like toxin-converting phages from *Escherichia coli* strains that cause hemorrhagic colitis or infantile diarrhea. *Science* 226 694–696. 10.1126/science.6387911 6387911

[B51] Oh J. Byrd A. L. Deming C. Conlan S. Kong H. H. Segre J. A. (2014). Biogeography and individuality shape function in the human skin metagenome. *Nature* 514 59–64. 10.1038/nature13786 25279917 PMC4185404

[B52] Penades J. R. Chen J. Quiles-Puchalt N. Carpena N. Novick R. P. (2015). Bacteriophage-mediated spread of bacterial virulence genes. *Curr. Opin. Microbiol.* 23 171–178. 10.1016/j.mib.2014.11.019 25528295

[B53] Pérez-Brocal V. García-López R. Nos P. Beltrán B. Moret I. Moya A. (2015). Metagenomic analysis of Crohn’s disease patients identifies changes in the virome and microbiome related to disease status and therapy, and detects potential interactions and biomarkers. *Inflamm. Bowel Dis.* 21 2515–2532. 10.1097/MIB.0000000000000549 26313691

[B54] Pérez-Brocal V. García-López R. Vázquez-Castellanos J. F. Nos P. Beltrán B. Latorre A. (2013). Study of the viral and microbial communities associated with Crohn’s disease: a metagenomic approach. *Clin. Transl. Gastroenterol.* 4 e36. 10.1038/ctg.2013.9 PMC3696940 23760301

[B55] Puig A. Araujo R. Jofre J. Frias-Lopez J. (2001). Identification of cell wall proteins of *Bacteroides fragilis* to which bacteriophage B40-8 binds specifically. *Microbiology* 147 281–288. 10.1099/00221287-147-2-281 11158345

[B56] Quiros P. Martinez-Castillo A. Muniesa M. (2015). Improving detection of Shiga toxin-producing *Escherichia coli* by molecular methods by reducing the interference of free Shiga toxin-encoding bacteriophages. *Appl. Environ. Microbiol.* 81 415–421. 10.1128/AEM.02941-14 25362055 PMC4272730

[B57] Reardon S. (2014). Phage therapy gets revitalized. *Nature* 510 15–16. 10.1038/510015a 24899282

[B58] Rodriguez-Brito B. Li L. Wegley L. Furlan M. Angly F. Breitbart M. (2010). Viral and microbial community dynamics in four aquatic environments. *ISME J.* 4 739–751. 10.1038/ismej.2010.1 20147985

[B59] Ross J. Topp E. (2015). Abundance of antibiotic resistance genes in bacteriophage following soil fertilization with dairy manure or municipal biosolids, and evidence for potential transduction. *Appl. Environ. Microbiol.* 81 7905–7913. 10.1128/AEM.02363-15 26341211 PMC4616940

[B60] Sanger F. Air G. M. Barrell B. G. Brown N. L. Coulson A. R. Fiddes C. A. (1977). Nucleotide sequence of bacteriophage phi X174 DNA. *Nature* 265 687–695. 10.1038/265687a0 870828

[B61] Scanlan P. D. Buckling A. (2012). Co-evolution with lytic phage selects for the mucoid phenotype of *Pseudomonas fluorescens* SBW25. *ISME J.* 6 1148–1158. 10.1038/ismej.2011.174 22189495 PMC3358020

[B62] Schmelcher M. Loessner M. J. (2014). Application of bacteriophages for detection of foodborne pathogens. *Bacteriophage* 4:e28137 10.4161/bact.28137 PMC3919822 24533229

[B63] Selva L. Viana D. Regev-Yochay G. Trzcinski K. Corpa J. M. Lasa I. (2009). Killing niche competitors by remote-control bacteriophage induction. *Proc. Natl. Acad. Sci. U.S.A.* 106 1234–1238. 10.1073/pnas.0809600106 19141630 PMC2633583

[B64] Srivastava A. S. Chauhan D. P. Carrier E. (2004a). In utero detection of T7 phage after systemic administration to pregnant mice. *Biotechniques* 37 81–83. 15283204 10.2144/04371ST04

[B65] Srivastava A. S. Kaido T. Carrier E. (2004b). Immunological factors that affect the in vivo fate of T7 phage in the mouse. *J. Virol. Methods* 115 99–104. 14656466 10.1016/j.jviromet.2003.09.009

[B66] Suttle C. A. (2005). Viruses in the sea. *Nature* 437 356–361. 10.1038/nature04160 16163346

[B67] Twort F. W. (1915). An investigation on the nature of ultra-microscopic viruses. *Lancet* 186 1241–1243. 10.1016/S0140-6736(01)20383-3 PMC2170983 20475326

[B68] Virgin H. W. (2014). The virome in mammalian physiology and disease. *Cell* 157 142–150. 10.1016/j.cell.2014.02.032 24679532 PMC3977141

[B69] Wagner J. Maksimovic J. Farries G. Sim W. H. Bishop R. F. Cameron D. J. (2013). Bacteriophages in gut samples from pediatric Crohn’s disease patients: metagenomic analysis using 454 pyrosequencing. *Inflamm. Bowel Dis.* 19 1598–1608. 10.1097/MIB.0b013e318292477c 23749273

[B70] Willner D. Furlan M. Haynes M. Schmieder R. Angly F. E. Silva J. (2009). Metagenomic analysis of respiratory tract DNA viral communities in cystic fibrosis and non-cystic fibrosis individuals. *PLoS ONE* 4:e7370 10.1371/journal.pone.0007370 PMC2756586 19816605

[B71] Zinder N. D. Lederberg J. (1952). Genetic exchange in *Salmonella*. *J. Bacteriol.* 64 679–699. 12999698 10.1128/jb.64.5.679-699.1952 PMC169409

